# Supplementary material for: Factors associated with difficulty of hospital acceptance of patients suspected to have cerebrovascular diseases: A nationwide population-based observational study
Source: PLoS One. 2021 Jan 12;16(1):e0245318. doi: 10.1371/journal.pone.0245318 (PMC7802939; doi:10.1371/journal.pone.0245318)
Supplement: Attachment — Submitted filename: PONE-D-20-03553_reviewer.docx [file pone.0245318.s001.docx]

In the present study, the authors aimed to investigate the factor associated with difficulty of hospital acceptance of patients suspected to have cerebrovascular diseases. When reviewing this manuscript, I am worrying that hospitals in Japan should be increasingly turning away sick people as the country struggles with surging coronavirus infections and its emergency medical system collapses.

Overall, this manuscript is well written. I only have the following minor comments:

1. In Line 103, “we excluded patients who were children (< 15 years old)…” And in Line 136, they said “…young people (< 15 years of age).” I suggest the authors to use a consistent term for < 15 years of age.
2. Line 124: In addition to the 4 categories of severity, can the authors accessed other variables (e.g. Japan Coma Scale) in their data set?
3. Line 129-130, they defined seven geographic regions (Hokkaido-Tohoku, Kanto, Chubu, Kansai, Chugoku, Shikoku, and Kyushu-Okinawa). But In Table 1, the name of the seven regions did not 100% match those written in Line 129-130. It’s better to revise it.
4. Line 164-168: “…the number of physicians per population in the area covered by each municipal fire department (<25 percentile, 25-75 percentile, 75 percentile≦), the proportion of emergency physicians to all physicians in the area (<25 percentile, 25-75 percentile, 75 percentile≦), the proportion of neurosurgeon to all physicians in the area (<25 percentile, 25-75 percentile, 75 percentile≦),…” Should “75 percentile≦” better be “76 percentile≦”? That’s because “25-75 percentile” already included 75.
5. As above, the use of “25-75 percentile” as reference was less intuitive and would made the results difficult to interpret. I suggest to use “76 percentile≦” as a reference.
6. Line 262: May add a limitation that the authors did not include national holidays, in addition to weekend, into analysis.

Keep fingers crossed and hope that Japan, as well as the whole world, will get through the tough situation right now.
