# Supplementary material for: Factors associated with difficulty of hospital acceptance of patients suspected to have cerebrovascular diseases: A nationwide population-based observational study
Source: PLoS One. 2021 Jan 12;16(1):e0245318. doi: 10.1371/journal.pone.0245318 (PMC7802939; doi:10.1371/journal.pone.0245318)
Supplement: Attachment — Submitted filename: 20201219 Response to Reviewers.docx [file pone.0245318.s003.docx]

**Re: PONE-D-20-03553R1**

Dear Editors and Reviewers:

Thank you for your thorough review of PONE-D-20-03553R	1, “Factors associated with difficulty of hospital acceptance of patients suspected to have cerebrovascular diseases: a nationwide population-based observational study”. Please find our responses to the Editors’ and Reviewers’ comments shown in **Bold** in the following response letter.

Sincerely,

Nobuhiro Sato

Reviewers' comments:

6. Review Comments to the Author

Please use the space provided to explain your answers to the questions above. You may also include additional comments for the author, including concerns about dual publication, research ethics, or publication ethics. (Please upload your review as an attachment if it exceeds 20,000 characters)

**Reviewer #1: The authors have addressed all my comments adequately.
The manuscript now looks nice.
I have no further comments.**

Thank you for your thorough reviews and comments.

**Reviewer #3: This study aimed to examine the predictors of difficulty of hospital acceptance of patients suspected to have cerebrovascular disease. It is unclear who and how to define those who were suspected to have cerebrovascular diseases? By the EMS personnel?**

We appreciate your suggestions. Hemiplegia, dysarthria, ataxia or severe headache were classified as cerebrovascular disease-related symptoms. The data were collected by EMS personnel, in cooperation with the physicians overseeing the patient’s care. We added information to the Methods section (Page 7, Para 108-109 and 111-112).

**Difficulty of acceptance was defined by having ≥4 phone calls by the EMS personnel to hospitals until acceptance. Who are the one responding to the phone calls? Triage nurses? Hospital administrators? Physicians?**

We thank the reviewer for the careful review of the manuscript. The person who responds to the phone calls by EMS personnel depends on the hospital. While physicians respond to the phone calls in some hospitals, nurses or the other staff respond in the other hospitals. We have added a description about this in the Introduction (Page 5, Para 69-70).

**Symptom presentation is a key factor to urge patients to call for help, same as healthcare professionals. Did the EMS personnel inform the one responding to the phone calls the symptoms of patients? Did they know that the patients are suspected to have cerebrovascular diseases?**

We appreciate your suggestions. Yes, EMS personnel informed medical staff responding to the phone calls about the symptoms which led EMS personnel to suspected cerebrovascular diseases according to EMS protocols.

**Mean age of physicians is one of the factor associated with difficulty of hospital acceptance, was this variable refer to those who refused to admit the patients or those who finally admitted the patients? did these physicians involved in refusing to admit patients?**

We appreciate reviewers' insightful comments. In this study, we analyzed hospital acceptance of each ambulance transport as opposed to acceptance of emergency admission to an inpatient ward from the emergency department. The variable mean age of physicians refers to the mean age of physicians in the area that each patient was transported from. Therefore, this is the mean age of physicians in the area who refused to accept the EMS transportation. We have added “in the area” to the Results and the Discussion (Page 12, Para 201, Page 17, Para 214 and 222-223, Page 20, Para 234, Page 21, Para 255, Page 23, Para 294).

**Minimal grammatical errors, but the manuscript requires some formatting to make it more readable, such as the “≦” should put in front of 75 percentile, Table 1, the fourth column heading should be ≥4**

We thank the reviewer for the careful review. We have corrected the typos.
